# Dosimetric performance evaluation of the Halcyon treatment platform for stereotactic radiotherapy: A pooled study

**DOI:** 10.1097/MD.0000000000034933

**Published:** 2023-09-08

**Authors:** Yangyang Huang, Zongwen Liu

**Affiliations:** a Department of Radiotherapy, the Second Affiliated Hospital of Zhengzhou University, Zhengzhou, China.

**Keywords:** dosimetric performance evaluation, gamma passing rates, Halcyon, plan complexity, plan quality, stereotactic radiotherapy

## Abstract

With the advancement of radiotherapy equipment, stereotactic radiotherapy (SRT) has been increasingly used. Among the many radiotherapy devices, Halcyon shows promising applications. This article reviews the dosimetric performance such as plan quality, plan complexity, and gamma passing rates of SRT plans with Halcyon to determine the effectiveness and safety of Halcyon SRT plans. This article retrieved the last 5 years of PubMed studies on the effectiveness and safety of the Halcyon SRT plans. Two authors independently reviewed the titles and abstracts to decide whether to include the studies. A search was conducted to identify publications relevant to evaluating the dosimetric performance of SRT plans on Halcyon using the key strings Halcyon, stereotactic radiosurgery, SRT, stereotactic body radiotherapy, and stereotactic ablative radiotherapy. A total of 18 eligible publications were retrieved. Compared to SRT plans on the TrueBeam, the Halcyon has advantages in terms of plan quality, plan complexity, and gamma passing rates. The high treatment speed of SRT plans on the Halcyon is impressive, while the results of its plan evaluation are also encouraging. As a result, Halcyon offers a new option for busy radiotherapy units while significantly improving patient comfort in treatment. For more accurate results, additional relevant publications will need to be followed up in subsequent studies.

## 1. Introduction

Stereotactic radiotherapy (SRT) is a technique that delivers high doses of radiation to tumors while preserving normal tissue function.^[1]^ SRT includes stereotactic radiosurgery (SRS), fractionated SRT, and stereotactic body radiotherapy (SBRT). In recent years, with the development of radiotherapy equipment, SRT has been moving toward large-scale applications.

The Halcyon, which entered clinical application in 2017, is characterized by its “fast” nature, with many features that distinguish it from the C-arm accelerators. First, it has an enclosed, ring-mounted gantry and a 6 MV flattening-free filter (FFF) beam that is used for treatment and imaging with Halcyon version 1.0. Halcyon 2.0 imaging no longer requires MV-X rays but adds a dedicated kV imaging system with greatly improved soft tissue resolution.^[2]^ It has a gantry speed of up to 4 rotations a minute. Since there is no field light, optical distance indicator or source-skin distance (SSD) measuring device, only the laser on the machine shell is used for patient positioning. Therefore, the accuracy of patient positioning depends on MV or kV imaging. Simple and clear designs named “one-step patient setup and verification” significantly reduces the daily setup time, which improves patient comfort and compliance. The Halcyon has no beam-shaping jaws, only stacked and staggered multileaf collimators (MLC) that form a maximum field of 28 cm × 28 cm at the isocenter. Each leaf is 1 cm wide, with 29 pairs of leaves in the proximal single-layer and 28 pairs in the distal single-layer, for a total of 114 leaves. The proximal single-layer is displaced laterally by 0.5 cm relative to the distal single-layer to reduce inter-leaf leakage. As a result, the Halcyon MLC leakage and transmission is less than 0.4% compared to 1.5% for the 6MV beam on TrueBeam (TB).^[3]^ At the same time, the less rounded leaves provide smaller penumbra with a smaller dosimetric leaf gap of 0.1 mm compared to 6 MV TB’s 1 mm. The maximum velocity of the leaves is 5.0 cm/s, which is twice as fast as the Varian C-arm accelerator MLCs.^[4]^ The average dose differences for the dual-layer, proximal single-layer and distal single-layer leaf sequences were −1.35%, −1.20%, and −1.34%, respectively. Therefore, the dosimetric parameters are comparable to the TB platform.^[5,6]^

The Halcyon is equipped with a treatment planning system (TPS) of Eclipse 15.1 or 15.6, the beam model in Eclipse is pre-defined, including percentage depth dose, profiles, and output factors.^[7,8]^ Halcyon 2.0 has 2 types of beams, the dynamic flattening beam and the FFF beam.^[9]^ Some authors^[10]^ conducted intensity-modulated radiotherapy and volumetric-modulated arc therapy (VMAT) delivery accuracy and mechanical quality assurance (QA) of Halcyon 2.0 following International Atomic Energy Agency TRS-398 and American Association of Physicists in Medicine TG-119 and found that it met all criteria. Lloyd et al^[11]^ concluded that the results of profile measurements at D_max_ for the Halcyon’s 6 MV-FFF beam varied by less than 0.5% with different active volumes of the Farmer ionization chambers. The results indicate that the Halcyon can perform reference dosimetry without a specialized chamber or lead filtering chamber and is well suited for resource-limited units. Bollinger et al^[12]^ studied the dosimetric features and limitations of the dynamic flattening beam on the Halcyon 2.0 and found that the transversal and radial profiles of ≥10 cm square fields were observed with <3% flatness under the conditions of 100 cm SSD and 10 cm water depth. The positioning accuracy of MLC, gantry angle, and couch shift were within 0.05 mm, 0.02°, and 0.03 mm, respectively.^[13]^ Therefore, the Halcyon demonstrates a potential of high delivery accuracy. Features of the Halcyon compared with C-arm accelerators are demonstrated in Table 1.

**Table 1:** Features of Halcyon comparing with C-arm accelerators.

Item	Halcyon	C-arm accelerators
Gantry	O-ring	C-arm
Enclosed gantry bore	Yes	No
Field light and optical distance indicator	No	Yes
Gantry rotation speed	4 rotations per minute	1 rotation per minute
Jaws	No	Yes
MLC layer	Dual-layer	Single-layer
MLC speed	5 cm/s	2.5 cm/s
IGRT	MV-CBCT or kV-CBCT	kV-CBCT and MV-EPID
Pre-defined beam model	Yes	No
Non-coplanar Beam	No	Yes
Maximum MLC-defined field	28 cm × 28 cm	40 cm × 40 cm
High treatment speed	Yes	No

CBCT = cone beam computed tomography, EPID = electronic portal imaging device, IGRT = image-guided radiotherapy, MLC = multileaf collimator.

Most Halcyon users are impressed with its treatment speed.^[3,14–20]^ Because of the distinctive features of Halcyon gantry rotation speed, dual-layer MLC, no jaw, no light field or SSD indicator, and the high requirements of SRT treatment, the dosimetric performance such as plan quality, plan complexity, and gamma passing rates of Halcyon SRT plans need to be explicitly studied. Plan evaluation is generally achieved by analyzing the TPS-calculated dose distribution. However, it is challenging to quantify 3-dimensional dose distributions and comprehensively analyze the spatial characteristics of dose distributions. We still need to gain the knowledge to personalize the prediction of clinical outcomes based on patient-specific characteristics. The plan quality requires the evaluation of various dosimetric indices derived from dose volume histogram (DVH)-based data, such as conformity index (CI), gradient index (GI), and homogeneity index (HI). In addition, the theoretical dose distribution is different from the actual dose distribution delivered to the patient due to uncertainties in dose calculations, errors in patient positioning, and changes in anatomical structures during the treatment course. Therefore, the dosimetric performance evaluation of SRT plans includes plan quality indices derived from DVH, such as CI, GI, and HI, as well as plan complexity and gamma passing rates.

This review analyzes the strengths and weaknesses of the Halcyon SRT plans to help radiotherapy practitioners better understand and implement the SRT plans on the Halcyon and to fully understand the effectiveness and safety of the Halcyon SRT plans.

## 2. Materials and methods

### 2.1. Literature search strategy

A PubMed search was performed to identify publications evaluating the dosimetric performance of SRT plans on Halcyon using the query strings Halcyon, SRS, SRT, SBRT, and stereotactic ablative radiotherapy. The SRT plans have the characteristics of a high fractionated dose (6–30 Gy/fraction), low fractions of treatments (1–5 fraction), high biological effect dose ≥ 100, high target conformality, and large off-target dose gradient compared with the conventional radiotherapy plans. Therefore, they have higher requirements for treatment equipment.

We searched PubMed for each of the following keywords/strings:

(Halcyon) AND SRT;

(Halcyon) AND SBRT;

(Halcyon) AND SRS;

(Halcyon) AND SABR.

The screening period was from November 19, 2017, to July 12, 2023. Studies were limited to the past 6 years and included only keywords in the title and/or abstract. The search was first conducted on November 19, 2022, and the supplemental search time is July 12, 2023.

### 2.2. Study selection

In order to reduce the bias related to the selective reporting of outcomes and increase the availability and accessibility of our article, 2 authors independently reviewed titles and abstracts to decide on the study inclusion.^[21]^ If titles and abstracts were considered relevant, the full text was retrieved. Only English publications were considered.

Publications were selected if they contained information about SRT plan quality evaluation (including dosimetric indices and organs at risk [OARs] evaluation) and provided a discussion and summary of one or more following medical physics components.

1. The Halcyon SRT plan complexity;
2. The Halcyon SRT plan gamma passing rates.

## 3. Results

### 3.1. Description of included studies and inclusion criteria

**Figure 1.**
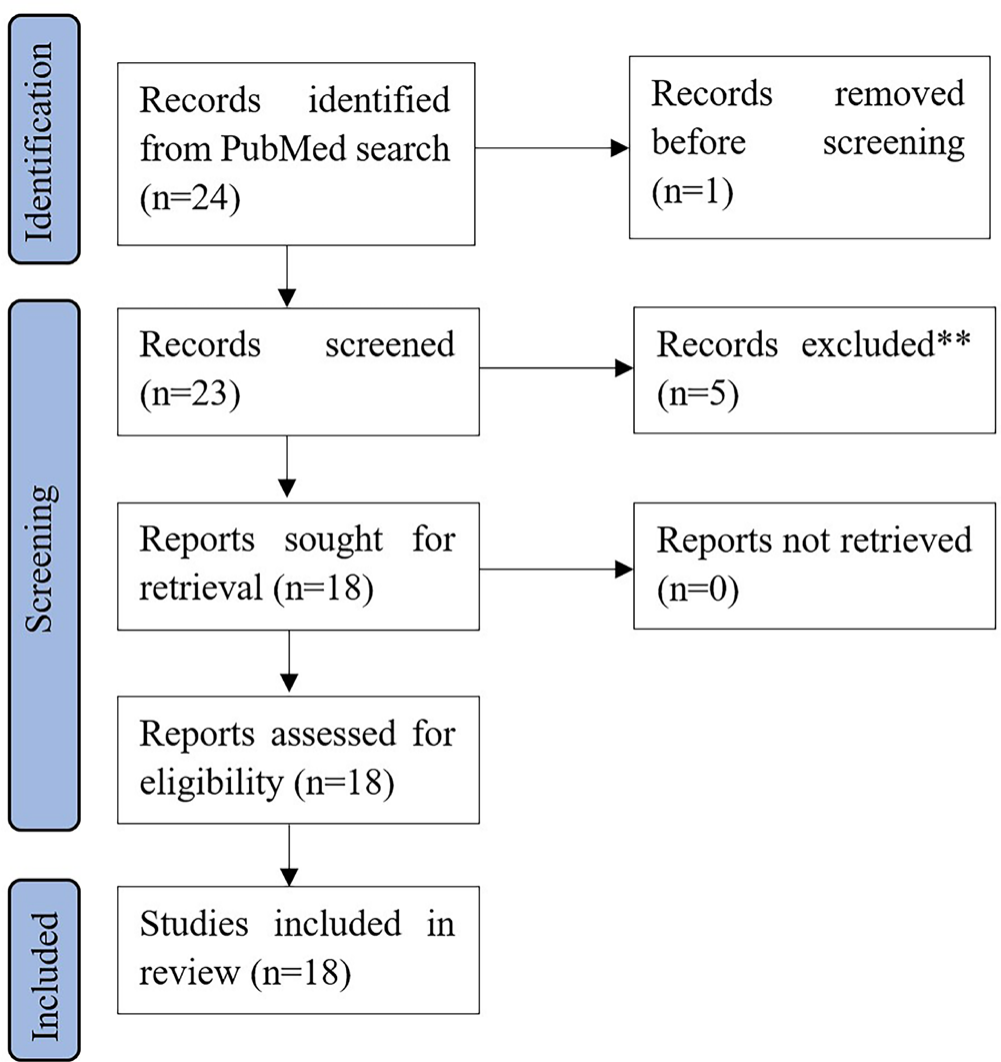
Numbers of included and excluded publications derived from the PubMed search related to the dosimetric performance evaluation of SRT plans on Halcyon. SRT = stereotactic radiotherapy.

Based on the PubMed search results, 24 publications were identified. The results are shown in Figure 1. Publications on the Halcyon accelerator appeared in 2018, as the production version of Halcyon was first used for patient treatment on September 14, 2017, at the University of Pennsylvania.^[8]^ It starts to increase significantly in the last 3 years. The number of publications related to 2023 was incomplete because the inclusion criteria were limited to July 12, 2023.

Out of 24 records, one was excluded due to duplication. Five publications were excluded from the 23 records screened for the following reasons: not relevant to the question addressed (#4) and not addressing Halcyon (#1). Of the 18 full-text publications assessed as eligible, 18 were selected based on the medical physics-related issues 1 and 2 described in the study selection paragraph in Section 2. Brief information about Halcyon plan quality, complexity, and gamma passing rates is presented in Table 2.

**Table 2:** Eighteen Halcyon SRT plan-related publications found in PubMed.

Ref.	Patients	Object	Tech.	Plan quality	Plan complexity	GPRs
Li et al^[22]^ 2019	10 patients with multiple intracranial metastases	HA 1.0 and 2.0, TB-HDMLC	VMAT-SRS	All of the targets had CIs < 2.5 with the majority of the targets’ CI below 2.0; TB achieved lower GI compared to HA	The total MUs were similar across all VMAT plans	NR
Reynoso et al^[23]^ 2020	7 healthy volunteers	HA 2.0, Edge	DCA-SRS	The lateral head flexion in HA showed an average improvement in CI of 7.3%, a decrease of 13% in R50, a decrease of 32% in R10, and a decrease of 7.8% in GM. The Edge plans showed an average improvement in CI of 3.0%, a decrease of 6.8% in R50, a decrease of 29% in R10, and a decrease of 5.0% in GM	NR	NR
Pokhrel et al^[15]^ 2021	8 patients with 2 to 3 abdominal/pelvic oligometastatic lymph nodes	HA 2.0, TB-Millenium 120 MLC	VMAT-SBRT	PTV CI was 1.05 ± 0.1 (0.99–1.15) for HA, 1.07 ± 0.1 (0.98–1.22) for TB; D2cm (%) was 50.5 ± 6.8 (39.5–58.7) for HA, 51.0 ± 6.9 (42.0–60.2) for TB (*P* > .05, *P* > .05)	Total MU was 2369 ± 772 (1476–3860) for HA, 2490 ± 741 (1576–3910) for TB; MF was 3.55 ± 0.93 (2.5–5.5) for HA, 3.74 ± 0.89 (2.7–5.6) for TB (*P* > .05, *P* > .05)	Pretreatment PD GPRs (2%/2 mm) (%) was 98.1 ± 1.6 (96.9–100.0) for HA, 97.7 ± 1.8 (96.6–100.0) for TB (*P* > .05)
Pokhrel et al^[19]^ 2021	10 prostate cancer patients	HA 2.0, TB-Millenium 120 MLC	VMAT-SBRT	PTV CI was 1.00 ± 0.05 (0.97–1.12) for HA, 1.01 ± 0.05 (0.96–1.11) for TB (*P* > .05); D2cm (%) was 63.00 ± 5.19 (53.90–70.80) for HA, 65.79 ± 6.68 (54.90–75.90) for TB (*P* = .006)	Mean total MU and MF were 2562 and 3.53 for HA vs 2666 and 3.68 for TB (*P* > .05, *P* > .05)	Pretreatment PD GPRs (2%/2 mm) (%) was 98.6 ± 1.5 (95.8–100.0) for HA, 98.3 ± 2.0 (98.3–100.0) for TB (*P* = .730)
Petroccia et al^[17]^ 2019	15 spine patients	HA 1.0, and 2.0, TB-HDMLC, TB-Millenium 120 MLC	VMAT-SBRT	All plans had similar CI and HI (HA 1.0: CI 1.0 ± 0.07, HI 0.29 ± 0.05; HA 2.0: CI 1.0 ± 0.06, HI 0.27 ± 0.05) as compared to TB (HD: CI 0.95 ± 0.03, HI 0.18 ± 0.06; Millennium: CI 0.96 ± 0.07, HI 0.21 ± 0.06) (all *P* > .05); the GI were 4.1 ± 0.7, 4.2 ± 0.7, 3.8 ± 0.7, and 3.6 ± 0.5, respectively for HA, Millennium, and HDMLC (all *P* < .05)	The average MCS were 0.28 ± 0.03, 0.18 ± 0.04, respectively for HA 2.0, and Millennium (*P* < .05)	With 2%/2 mm criterion, All plans for all modalities met the criteria of above 90%; the GPRs for HA 1.0 and 2.0 were 98.8 ± 0.2%, 96.9 ± 2.0%, respectively (*P* > .05)
Visak et al^[24]^ 2021	20 lung cancer SBRT	HA 1.0, and 2.0, TB	VMAT-SBRT	CI was 1.01 ± 0.02 for k-HA, 1.01 ± 0.04 for c-TB (*P* > .05); HI was 1.19 ± 0.02 for k-HA, 1.14 ± 0.04 for c-TB (*P* < .001); GI was 4.22 ± 1.2 for k-HA, 4.65 ± 1.2 for c-TB (*P* = .005)	Total MU was 4076 ± 608 for k-HA, 3137 ± 873 for c-TB (*P* < .001); MF was 4.08 ± 0.6 for k-HA, 3.14 ± 0.9 for c-TB (*P* < .001)	NR
Pokhrel et al^[20]^ 2020	15 lung SBRT patients	HA 2.0, TB-Millenium 120 MLC	VMAT-SBRT	PTV CI was 1.01 ± 0.03 (0.98–1.09) for HA, 1.00 ± 0.03 (0.97–1.07) for TB (*P* > .05); HI was 1.24 ± 0.03 (1.2–1.3) for HA, 1.23 ± 0.04 (1.2–1.4) for TB (*P* > .05); GI was 4.64 ± 1.1 (3.5–7.6) for HA, 4.34 ± 0.9 (3.40–7.07) for TB (*P* = .004); D2cm (%) was 52.1 ± 5.5 (44.9–62.3) for HA, 51.7 ± 4.5 (44.6–61.6) for TB (*P* > .05)	Total MU was 3128 ± 722 (1976–5139) for HA, 3450 ± 807 (2656–5945) for TB (*P* = .034); MF was 3.13 ± 0.72 (1.98–5.14) for HA, 3.45 ± 0.81 (2.66–5.95) for TB (*P* = .0034)	With 2%/2 mm criterion, the GPRs (%) for HA and TB were 94.4 ± 2.1 (93.5–97.8), 93.0 ± 2.5 (91.4–96.5), respectively (*P* = .041)
Pokhrel et al^[16]^ 2022	6 patients with double-vertebral segments on thoracic and/or lumber spine	HA 2.0, TB-Millenium 120 MLC	VMAT-SBRT	PTV CI was 1.05 ± 0.06 (0.99–1.15) for HA, 1.04 ± 0.04 (1.00–1.10) for TB (*P* > .05); HI was 1.20 ± 0.01 (1.19–1.23) for HA, 1.18 ± 0.03 (1.16–1.22) (*P* > .05); GI was 3.90 ± 0.5 (3.3–4.4) for HA, 3.90 ± 0.30 (3.6–4.5) for TB (*P* > .05); D2cm (%) was 50.2 ± 4.0 (45.5–55.2) for HA, 50.5 ± 3.7 (45.6–54.0) for TB (*P* > .05)	Total MU was 3892 ± 880 (2736–5134) for HA, 4023 ± 659 (2888–4852) for TB; MF was 5.6 ± 1.3 (3.9–7.3) for HA, 5.7 ± 0.9 (4.1–6.9) for TB	With 2%/2 mm criterion, the GPRs (%) for HA and TB were 99.6 ± 0.3 (99.3–100.0), 96.8 ± 1.2 (94.8–98.1), respectively (*P* < .05)
Li et al^[25]^ 2021	20 patients with a single-level spine metastasis	HA, TB STx	VMAT-SBRT	pCI was 0.77 ± 0.1 for HA, 0.83 ± 0.1 for TB (*P* < .05); pGI was 4.06 ± 0.9 for HA, 3.85 ± 0.7 for TB (*P* > .05); HI was 1.34 ± 0.1 for HA, 1.31 ± 0.1 for TB (*P* > .05)	Total MU was 4775 ± 1591 for HA, 5463 ± 2155 for TB (*P* > .05)	NR
Sarkar et al^[26]^ 2021	9 early stage non-small cell lung cancer cases	HA, Novalis Tx	NR	NTX and HA plans had the same PTV coverage of 98% volume receiving 97% of prescription dose, and the global dose maximums were 106.6% and 110.1%, respectively; V20Gy for normal lung were 14.9% and 19.7%, respectively	The MUs were 6556.8 for NTX, and 3696 MUs for HA	NR
Pokhrel et al^[14]^ 2022	16 two-lesion lung cases	HA 2.0, TB-Millenium 120 MLC	VMAT-SBRT	PTV CI was 1.04 ± 0.09 (0.93–1.21) for HA, 1.06 ± 0.1 (0.89–1.29) for TB (*P* = .054); HI was 1.22 ± 0.05 (1.14–1.31) for HA, 1.24 ± 0.05 (1.15–1.27) (*P* = .091); GI was 5.13 ± 1.13 (3.69–8.12) for HA, 5.25 ± 1.06 (3.60–7.64) for TB (*P* = .136); D2cm (%) was 54.1 ± 4.3 (47.2–65.8) for HA, 54.8 ± 5.9 (47.6–68.9) for TB (*P* = .869)	Total MU per fraction was 4532 ± 890 (2852–6007) for HA, 4052 ± 702 (2770–5439) for TB (*P* = .041); MF was 4.53 ± 0.89 (2.85–6.01) for HA, 4.05 ± 0.71 (2.77–5.44) for TB (*P* = .043)	With 2%/2 mm criterion, the PD GPRs (%) for HA and TB were 98.45 ± 0.99 (97.9–100), 98.9 ± 0.85 (98.1–100), respectively (*P* = .064)
Altundal et al^[27]^ 2020	15 prostate cancer patients	HA 2.0, CyberKnife G4	VMAT-SBRT for HA,	The CI, pCI and HI were 1.02 ± 0.01, 0.91 ± 0.01, 1.06 ± 0.01, respectively, for HA; there were statistically significant improvements (*P* < .05) on the CI, pCI and HI for HA	For the 3-Arc HA plans, the average total MU per fraction was 3093.9 ± 134.9	The GPR for HA was 99.75 ± 0.08% with a 2%/2mm criterion and a 10% threshold
Wada et al^[28]^ 2021	50 cases of lung cancer	HA, TB	3D-CRT-, IMRT-, and VMAT-SBRT	When compared with clinical TB plans’ CI of 1.22 ± 0.09, the CI was 1.16 ± 0.05 (*P* = .01) for KBP_open_-HA, 1.14 ± 0.04 (*P* = .003) for KBP_open_-TB; compared with clinical TB plans’ HI of 23.98 ± 5.40, HI was 22.72 ± 1.97 (*P* = .36) for KBP_open_-HA, 21.96 ± 1.71 (*P* = .16) for KBP_open_-TB	When compared with clinical TB plans’ MU of 3527.94 ± 655.43, the MU was 3311.17 ± 290.15 (*P* = .57) for KBP_open_-HA, 4176.44 ± 466.55 (*P* = .003) for KBP_open_-TB	NR
Pokhrel et al^[29]^ 2021	15 lung SBRT patients	HA 2.0, TB-Millenium 120 MLC	VMAT-SBRT	NR	NR	NR
Roover et al^[30]^ 2021	20 prostate cancer patients	HA 2.0, TB-Millenium 120 MLC, TB STx-HDMLC	VMAT-SBRT	The median (Range) of CI was 1.16 (1.08, 1.27) for HA 2.0, 1.13 (1.08, 1.24) for TB-HDMLC, 1.15 (1.08, 1.24) for TB-Millenium; The median (Range) of D2cm (%) was 56.3 (52.4, 59.8) for HA 2.0, 53.8 (51.7, 56.7) for TB-HDMLC, 54.8 (52.6, 58.2) for TB-Millenium	The median (Range) of MU was 2255 (1938, 2798) for HA 2.0, 2605 (2407, 2844) for TB-HDMLC, 2614 (2341, 2809) for TB-Millenium	With 2%/2 mm criterion, the median (Range) of GPR (%) was 99.7 (96.4, 100.0) for HA 2.0, 99.2 (97.3, 100.0) for TB-HDMLC, 99.7 (97.8, 100.0) for TB-Millenium
Wang et al^[31]^ 2022	61 patients	HA 2.0	IMRT/VMAT/SRT	NR	NR	With 2%/2 mm criterion, the average GPRs were ArcCHECK at 96.4% and PD at 96.7%, respectively
Byrne et al^[32]^ 2022	20 lung tumor patients, 20 brain tumor patients	HA 2.0, Ethos	VMAT-SRT	The median (95% Confidence Interval) pCI were 0.89 (0.84–0.93) for HA 2.0 in lung plans, 0.89 (0.79–0.91) for HA 2.0 in brain plans	The median (95% Confidence Interval) MU/cGy were 2.81 (2.05–3.91) for HA 2.0 in lung plans, 2.26 (1.65–3.12) for HA 2.0 in brain plans	With 3%/2 mm criterion, the median (95% Confidence Interval) GPRs were 99.9% (99.4%–100.0%) for HA 2.0 in lung plans, 99.4% (98.1%–99.9%) for HA 2.0 in brain plans
Pokhrelet al^[3]^ 2023	10 APBI patients	HA 2.0, TB-Millenium 120 MLC	VMAT-SBRT	PTV D95% coverage (Gy) were 25.72 ± 0.34 (25.72–26.07) for HA, 25.73 ± 0.42 (24.71–26.03) for TB-Millenium (*P* = .954)	Modulation factors were 2.28 ± 0.40 (1.16–2.87) for HA, 1.77 ± 0.22 (1.35–1.97) for TB-Millenium (*P* = .001)	With 3%/2 mm criterion, the GPRs (%) were 99.6 ± 0.39 (98.8–100) for HA 2.0, 97.85 ± 2.63 (91.5–100) for TB-Millenium (*P* = .001)

3DCRT = 3 dimensional conformal radiotherapy, APBI = accelerated partial breast irradiation, CI = conformity index, c-TB = clinical planning on TrueBeam, D2cm = the maximal dose at 2 cm away from the PTV in any direction, DCA = dynamic conformal arc, GI = gradient index, GM = gradient measure, GPR = gamma passing rate, HA = Halcyon, HDMLC = high definition multileaf collimator, HI = Homogeneity index, IMRT = intensity-modulated radiotherapy, KBP_open_-HA = knowledge-based planning for new patient cases on Halcyon, KBP_open_-TB = knowledge-based planning for new patient cases on TrueBeam, k-HA = knowledge-based planning on Halcyon, MCS = modulation complexity score, MF = modulation factor, MUs = monitor units, NR = not reported, NTX = Novalis Tx, pCI = Paddick conformity index, PD = portal dosimetry, pGI = Paddick gradient index, PTV = planning target volume, R10 = ratio of the volume of the 10% of prescription isodose curve to PTV, R50 = ratio of the volume of the 50% of prescription isodose curve to PTV, SBRT = stereotactic body radiotherapy, SRS = stereotactic radiosurgery, SRT = stereotactic radiotherapy, TB = TrueBeam, V20Gy = the percentage of 20 Gy isodose account for the whole volume, VMAT = volumetric-modulated arc therapy.

### 3.2. Plan quality

The dosimetric performance evaluation of SRT plans on Halcyon requires plan quality evaluation to overcome the planning uncertainties due to planners’ subjective tendencies and lack of ability. The plan evaluation includes slice-by-slice checking the relationship between isodose lines and the target volume/OARs, evaluating the gradient measure (GM), the maximal dose at 2 cm away from the planning target volume in any direction (D2cm) that comes with the TPS, and DVH-based dose coverage, CI, GI, and HI dosimetric indices.

The slice-by-slice checking of the relationship between isodose lines and the target volume/OARs can provide the observer with 3-dimensional dose information about the target volume and OARs. The checking can identify where there are cold spots and hot spots and whether modifications are needed, thus further improving the quality of SRT plans.^[22]^

The TPS comes with GM, D2cm, and other indices that can be read directly in the software. Among the DVH-based dosimetric indices, the target dose coverage can be visualized on the DVH and is, therefore, the most commonly used. There are 2 standard definitions of dose coverage, the radiation therapy oncology group (RTOG) method^[23]^ and the International Commission on Radiation Units and Measurements method.^[24]^ A qualified CI guarantees plan quality among the dosimetric indices such as CI, GI, GM, D2cm, and HI. GI, GM, and D2cm take into account the dose fall-off outside the target and are helpful for OARs sparing, while HI is gradually marginalized in the quality evaluation of SRT plans. The CI of RTOG^[25]^ did not consider the location and shape of the prescriptive isodose line relative to the targets. To overcome the limitation, Paddick et al^[26]^ proposed the Paddick CI, which combines conformity and dose coverage and better solves the problem of the false perfect score.^[22]^ The steep dose fall-off is essential to reduce the dose of OARs adjacent to the targets, and GI, GM, and D2cm can quantify this tendency.^[26–28]^ HI describes the degree of dose uniformity within the target.^[1]^ The pursuit of dose uniformity within the target will increase the dose of OARs and reduce the protection intensity of OARs by TPS. Therefore, when the targets of SRT plans do not contain functional tissue to be spared, the maximum target dose should be set carefully.^[29]^ The specific definitions of dose coverage, CI, GI, GM, D2cm, and HI are shown in Table 3 below.

**Table 3:** Definitions of standard plan quality evaluation indices.

Dosimetric indices	Ref.	Formulation and parameter description
Dose coverage	RTOG^[22]^	Dose coverage = *I*_min_/RI Where, *I*_min_ is the minimum isodose surrounding the target volume, RI is the reference isodose
	ICRU^[23]^	Dose coverage = TV_PIV_/TV × 100% Where, TV_PIV_ is the volume of the target covered by the prescription isodose, TV is the target volume
CI	RTOG^[24]^	CI = PIV/TV Where, PIV is the prescription isodose volume, TV is the target volume
	Paddick et al^[25]^	CI = TV2 PIV/(TV × PIV) Where, TV_PIV_ is the volume of the target covered by the prescription isodose, TV is the target volume, PIV is the prescription isodose volume
GI	Paddick et al^[25]^	GI = PIV_50%_/PIV Where, PIV is the prescription isodose volume, PIV_50%_ is half of the prescription isodose volume
	Mayo et al^[26]^	GI = 50%/(R_eff, RX_- R_eff, 50%RX_) Where, R_eff, RX_ is effective radii of 100% isodoses, R_eff, 50%RX_ is effective radii of 50% isodoses
D2cm	RTOG^[27]^	Maximum dose to any point 2 cm away from the target margin
GM	Varian^[29]^	GM = R_50%Dp_-R_Dp_ Where, R_50%Dp_ is the equivalent sphere radius of 50%prescription dose, R_Dp_ is the equivalent sphere radius of prescription dose
HI	RTOG^[22]^	HI = *I*_max_/RI Where, *I*_max_ is the maximum isodose in the target, RI is the reference isodose
	ICRU^[30]^	HI = (D_2%_–D_98%_)/D_50%_ Where, D_x%_ is the minimal dose to the x% highest irradiated target volume

CI = conformity index, GI = gradient index, GM = gradient measure, HI = homogeneity index.

Most authors analyzed the plan quality of Halcyon by comparing it with TB, a state-of-the-art C-arm accelerator of Varian (Varian Medical Systems, Palo Alto, CA), which represents the latest achievement in radiotherapy equipment.^[30,31]^ Halcyon has a strong dose carving capability for SRT plans. The stacked and staggered MLC can produce highly conformal dose distribution to targets, comparable intermediate dose spillage, and similar doses to adjacent OARs compared to TB. See Table 2 for details. Pokhrel et al^[3,14–16,19]^ conducted VMAT-SBRT technique using Halcyon 2.0 and designed plans for abdominal/pelvic oligometastatic lymph nodes, double-vertebral segments, single-isocenter/2-lesion lung, prostate and accelerated partial breast irradiation patients respectively. They compared the CI, HI, GI, D2cm, and OARs of these plans with those of TB Millenium 120 MLC in the same group of patients. The differences between these indices were mainly not statistically significant. Petroccia et al^[17]^ studied the Halcyon VMAT-SBRT for vertebral metastases, using plan quality indices such as target coverage, HI, CI, and GI. They found that the Halcyon can generate comparable and clinically equivalent spine SBRT plans to TB plans with less rapid dose fall-off out of the targets. Li et al^[32]^ designed 20 cases of plans with a single-level spine metastasis located between the T7 and L5 vertebrae near the spinal canal, and the Halcyon VMAT-SBRT plans achieved similar target coverage (Halcyon: 92.3 ± 3.0% vs TB: 92.4 ± 3.3%, *P* = .82) and CI (Halcyon: 1.0 ± 0.1 vs TB: 1.1 ± 0.2, *P* = .12) compared to the TB VMAT-SBRT plans. Despite the data were not statistically significant (*P* > .05), the GI (Halcyon: 3.96 ± 0.8 vs TB: 3.85 ± 0.7) of Halcyon plans were higher. Roover et al^[33]^ compared the Halcyon 3-arc VMAT-SBRT plans with the TB 2-arc VMAT-SBRT plans in 20 prostate cancer cases and found that the Halcyon 3-arc plans showed higher planning target volume coverage (D_99%_) of the seminal vesicle, along with reduced high-dose spillage of the bladder (V_37Gy_) and urethral (D_0.035cc_). Altundal et al^[34]^ compared 20 prostate patients’ SBRT plans of Halcyon 2.0 and CyberKnife and found that all Halcyon SBRT plans met the goals and constraints of the RTOG 0938 protocol, with improved CI and target dose coverage, reduced maximum dose in the skin and urethra compared to the CyberKnife plans. Comparison of Halcyon SRT plans with their counterparts in high-end devices such as TB led to the optimistic conclusion that the Halcyon VMAT-SRT plans were dosimetrically equivalent to TB plans, and can achieve better dosimetric performance for some treatment sites.

The ability of Halcyon to treat patients rapidly is an undisputed fact. Meanwhile, the rapid generation of high-quality Halcyon plans is breaking through. Automated planning is an essential complement to the “rapid treatment matrix” of Halcyon and is the most feasible direction for rapid Halcyon planning. Wada et al^[35]^ used a simplified knowledge-based planning model to design the Halcyon VMAT-SBRT plans for lung cancer, and the plan quality was clinically acceptable. In order to validate the feasibility of Halcyon-based rapid SBRT plans for central lung cancer, Visak et al^[36]^ designed lung cancer SBRT plans using the KBP model, and new plans similar or better in dosimetry to the TB plans were obtained within 30 min, where the CI difference was not significant (*P* > .05). Halcyon SBRT plans had a mean lower GI of 0.43 (*P* = .006), an increase in maximum target dose of 2.9 Gy (*P* < .001), and a mean reduction in lung mean lung dose of 0.10 Gy (*P* = .004).

However, there is room for continued improvement of the Halcyon over conventional high-end C-arm accelerators. Because of the ring gantry, Halcyon can provide translational but not rotational couch corrections, allowing only 3 degrees of freedom (DoF) corrections. However, C-arm linear accelerators can provide translational and rotational couch corrections, allowing 6 DoF corrections.^[32]^ Hence, the Halcyon linear accelerator cannot provide non-coplanar fields, which may lead to limitations in SRT implementations.^[37,38]^ However, lateral head flexion can overcome the inability to design non-coplanar SRS plans and considerably increase beam entrance angles. It thus improves plan conformity and normal tissue sparing. Reynoso et al^[39]^ did the research and found that when comparing the head flexion technique with a fully coplanar geometry, the Halcyon SRS plans showed an average improvement in CI of 7.3% (1.46 ± 0.25 vs 1.36 ± 0.28), a decrease of 13% in intermediate dose fall-off (5.46 ± 1.14 vs 4.78 ± 1.12), a decrease of 32% in low dose spillage (85.7 ± 20.3 vs 58.2 ± 15.1), and a decrease of 7.8% in gradient measure (0.53 ± 0.05 vs 0.49 ± 0.04). Li et al^[40]^ conducted a retrospective study of ten patients with multiple intracranial metastases, each with 6 to 10 targets with a volume of 0.11 to 8.57 cc and a prescribed dose of 15 to 24 Gy. They compared CI, GI, and OARs from Halcyon and TB plans in single-isocenter VMAT-SRS treatment. They found that for targets > 1 cm in diameter, Halcyon could produce a CI value similar to TB with reduced low-dose leakage to normal brain tissue. The GI and intermediate dose fall-off for the Halcyon plans were inferior to the TB plans, probably because of the inability of Halcyon to achieve non-coplanar geometry and 1 cm wide MLC leaves.^[17]^

In conclusion, the quality of the Halcyon SRT plans is comparable with the TB SRT plans, which, combined with its fast treatment speed, thus significantly improves patient comfort and clinic workflow. It is an option for busy radiotherapy centers.

### 3.3. Plan complexity

Plan evaluation includes plan complexity. Plan complexity is a corollary to the stringent requirements of SRT plans for dose coverage, CI, and GI. Meanwhile, SRT plans always have smaller targets. All the above factors yield results such as smaller and less regular beam apertures, more significant tongue-and-groove effects, more extensive modulation of machine parameters, and more monitor units (Mus).^[41–43]^ In the planning process, the plan complexity increases as the minimum segment area decreases and the number of optimization iterations and the maximum number of segments increases.^[42]^ Therefore, excessive complex plans can lead to dose calculation and delivery uncertainties^[44–47]^ and a decrease in treatment efficiency.^[40]^ In addition, patient respiratory movements and gastrointestinal motility can also affect the accuracy of dose delivery by producing effects such as the Interplay effect.^[48]^ In conclusion, plan complexity needs to be measured and controlled in advance for dose delivery accuracy and treatment efficiency.

Many plan complexity indices are interrelated, and most can be used to describe different aspects and sources of plan complexity.^[49]^ The leading indices are MUs, the ratio of total number of MUs per fraction to the prescription dose in cGy defined as the modulation factor (MF), aperture shape controller, modulation complexity score (MCS), etc. The smaller the value of all the above indices, the lower the complexity of the plans. Eclipse TPS includes aperture shape controller tools^[50]^ that can limit the complexity during plan optimization, partly by reducing the number of total MUs, stabilizing the gantry rotation speed, reducing the number of small-area segments, reducing the number of irregular fields, etc. Quintero et al^[51]^ considered that the complexity of plans is positively related to the number of MUs. McNiven et al^[52]^ and Masi et al^[53]^ found that smaller segments and irregular fields increase the beam-on time and dose calculation error, and designed the MCS index to analyze plan complexity. Different indices of plan complexity focus on different aspects of plan composition, ranging from studies of MF,^[42,47]^ to analyses of field size and irregularity,^[42,54]^ to studies of dose rates, the maximum range of MLC leaf motion, and gantry rotation speed.^[55,56]^

Most authors analyzing Halcyon’s plan complexity compared it with TB, and many obtained favorable results. Pokhrel et al^[16]^ using MD Anderson’s single-isocenter/multi-target (lung/spine targets) thorax phantom, compared the MUs, MF, and other indices of Halcyon and TB plans when performing double-vertebral segments VMAT-SBRT treatment. They found that the MUs and MF of Halcyon plans were reduced by 130 MU and 0.2, respectively. In the VMAT-SBRT treatment of lung cancer, Pokhrel et al^[20]^ found that the MUs per fraction (Halcyon: 3128 ± 722 vs TB: 3450 ± 807, *P* < .05) and MF (Halcyon: 3.13 ± 0.72 vs TB: 3.45 ± 0.81, *P* < .05) of Halcyon plans were significantly lower than those of TB plans. Li et al^[32]^ analyzed 20 cases of VMAT-SBRT plans with a single-level spine metastasis located between the T7 and L5 vertebrae near the spinal canal and found that the MUs of the Halcyon plans (Halcyon: 4998 ± 1688 vs TB: 5463 ± 2155, *P* = .09) were comparable to those of the TB plans. Li et al^[40]^ showed the same findings in VMAT-SRS treatment of multiple brain metastases with a single isocenter.

However, many authors have also argued that the Halcyon SRT plans are more complex than the TB’s. Petroccia et al^[17]^ found that the TB Millennium 120 MLC SRT plans had a lower average MCS value of 0.18 ± 0.04 compared to the Halcyon 2.0 plans of 0.28 ± 0.03. Pokhrel et al^[14]^ found in the Halcyon 2.0 treatment of single-isocenter/2-lesion lung in the VMAT-SBRT plans that the Halcyon plans had an average increase in MUs and MF of 480 and 0.48, respectively, compared to the TB plans.

Overall, literature supporting the Halcyon SRT plans as less complex than the TB SRT plans dominates. In clinical planning, planners should minimize plan complexity while meeting clinical dose requirements and dose delivery accuracy to reduce uncertainty in overall plan execution. Information on plan complexity helps planners make trade-offs between dosimetric indices and plan complexity. The plan complexity can also be used as a validation tool to reduce the QA workload. For example, low complexity plans may require little validation,^[57]^ which is essential for implementing online adaptive radiotherapy and can significantly reduce the time spent on adaptive processes.^[58]^

### 3.4. Gamma passing rates

Plans are designed to be used in radiotherapy practice, and there are often discrepancies between the theoretical dose distribution and the actual dose distribution.^[59]^ The gamma passing rate is a parameter used to describe the dose discrepancy between theory and practice.^[60]^ Gamma passing rates are collected at criteria of *x*%/*y* mm (*x, y* = 1, 2, 3) with a low threshold of 5% to 20%.^[61]^ Standard collection tools of gamma passing rates are 2D or 3D matrix devices and tools that come with the accelerator manufacturer, such as Varian portal dosimetry (PD). The patient anatomical site variations and structural differences,^[62]^ TPS algorithms,^[63]^ plan complexity,^[47]^ and device accuracy^[64]^ will likely affect gamma passing rates. Halcyon’s gantry rotation speed and MLC motion speed are faster than C-arm accelerators, so the dose delivery accuracy of SRT plans is a problem worth studying.

Pokhrel et al^[14–16,19,20]^ conducted intensive multi-disease SRT plan delivery accuracy studies with the Halcyon platform from 2021 to 2022. The experimental results demonstrate that the delivery accuracy of SRT plans on the Halcyon platform can reach the level of TB. In Pokhrel et al 2021,^[15]^ they performed VMAT-SBRT treatment on 8 patients with 2 to 3 abdominal/pelvic oligometastatic lymph nodes, and the plan PD results showed a high gamma pass rate of 98.1 ± 1.6% at the 2%/2 mm criterion. In the same year,^[19]^ they performed VMAT-SBRT treatment on ten prostate cancer patients. The dose delivery accuracy of the Halcyon SRT plans and the corresponding TB SRT plans were 98.6 ± 1.5% (range 95.8–100.0%) and 98.3 ± 2.0% (range 95.3–100%), respectively, and there was no statistical difference (*P* > .05). Also, in Pokhrel et al 2021,^[20]^ they performed the same treatment on 15 lung cancer cases. They found that the gamma passing rates with a 2%/2 mm global gamma criterion with a low dose threshold of 10% of the TB plans and the corresponding Halcyon plans were 93.0 ± 2.5% (ranged, 91.4–96.5%) and 94.4 ± 2.1% (ranged, 93.5–97.8%) on average, respectively. Halcyon plans showed significantly better gamma passing rates (*P* = .041) compared to those of TB plans. In Pokhrel et al 2022,^[16]^ they replanned 6 patients with double-vertebral segments on the thoracic and/or lumber spine. The average results of patient-specific portal dosimetry QA performed on the Halcyon showed a mean gamma passing rate of 99.6% compared to a mean 96.8% of the SBRT-specific TB for 2%/2 mm criterion, indicating a more accurate treatment outcome with the Halcyon. Also, in Pokhrel et al 2022,^[20]^ they designed 16 pairs of single-isocenter/2-lesion lung VMAT-SBRT plans. Each plan’s delivery accuracy was assessed by PD with a criterion of 2%/2 mm and a low-dose threshold setting of 5%. The mean gamma passing rate was 98.9 ± 0.85% (range: 98.1–100%), and 98.45 ± 0.99% (range: 97.9–100%) for the clinical TB plans and the corresponding Halcyon plans, respectively, and *P* > .05 demonstrated similar treatment delivery accuracy. In Pokhrel et al 2023,^[3]^ they selected 10 accelerated partial breast irradiation VMAT-SBRT plans. Each plan’s delivery accuracy was assessed by PD with a criterion of 3%/2 mm and a low-dose threshold setting of 10%. The mean gamma passing rate (%) was 99.6 ± 0.39 (98.8–100) for Halcyon 2.0, 97.85 ± 2.63 (91.5–100) for TB-Millenium, *P* = .001 demonstrated better treatment delivery accuracy for Halcyon 2.0.

Based on 15 spine SBRT plans, Petroccia et al^[17]^ found that the gamma passing rates for Halcyon 1.0 and 2.0 with 2%/2 mm criterion were 98.8 ± 0.2% and 96.9 ± 2.0%, respectively, and no statistical difference in gamma passing rates was observed between Halcyon and TB plans. Altundal et al^[34]^ analyzed 15 prostate cancer patients and found that Halcyon plans’ average gamma passing rate was 99.75 ± 0.08 with a 2%/2 mm criterion and a 10% threshold. With the same criterion, Roover et al^[33]^ used portal dosimetry to collect the gamma passing rates of 20 prostate cancer patients of the TB HD-MLC VMAT-SBRT plans and the Halcyon VMAT-SBRT plans. The results were 99.2% (range: 97.3, 100.0%) and 99.7% (range: 96.4, 100.0%), respectively. Wang et al^[65]^ analyzed 61 cases of Halcyon plans and found that the average gamma passing rate with a 2%/2 mm criterion for intensity-modulated radiotherapy/VMAT/SRT plans was 96.4% for ArcCHECK and 96.7% for portal dosimetry, respectively. With the 3%/3 mm criterion, the gamma passing rate of all plans was 99.1 ± 1.1% for ArcCHECK. All plans were within 3% of point dose measurements. Based on the results of Byrne et al,^[66]^ the median (95% Confidence Interval) gamma passing rates were 99.9% (99.4–100.0%) for Halcyon 2.0 in lung plans, 99.4% (98.1–99.9%) for Halcyon 2.0 in brain plans with 3%/2 mm criterion.

By comparing the gamma passing rates with those of TB, it was found that the Halcyon SRT plans frequently have higher values, which means that the Halcyon SRT plans have a higher plan deliverability.

## 4. Conclusions

The application of Halcyon is exciting, but 3 critical issues must be highlighted. First, Halcyon’s SRT plan is good enough. The most considerable direction of improvement for Halcyon in the future lies in developing an automatically rotational 6-DOFs couch and a higher dose rate. Second, this review does not refer to planning issues. Indeed, planning issues are an essential aspect of SRT practice. Third, more relevant publications need to be tracked and studied later to get more results.

This review indicated Halcyon could generate SRT plans with higher plan quality, less plan complexity, and higher gamma passing rates compared with those of the TB. The treatment time for Halcyon is significantly faster than TB, despite the former being currently limited by 800 MU/min dose rate. However, in some cases, TB is still preferred over Halcyon for the 6-DoFs couch and automatic rotational correction capabilities for image-guided radiotherapy. After overcoming the above limitations, the novel Halcyon platform is likely a more attractive option for SRT practice.

## Author contributions

**Conceptualization:** Yangyang Huang.

**Formal analysis:** Yangyang Huang.

**Funding acquisition:** Zongwen Liu.

**Investigation:** Yangyang Huang.

**Methodology:** Yangyang Huang.

**Project administration:** Zongwen Liu.

**Resources:** Zongwen Liu.

**Supervision:** Zongwen Liu.

**Visualization:** Yangyang Huang.

**Writing – original draft:** Yangyang Huang.

**Writing – review & editing:** Yangyang Huang, Zongwen Liu.
